# Volume-activated chloride channel exists in CD133+ lung cancer stem cells

**DOI:** 10.1186/1749-8090-8-S1-P135

**Published:** 2013-09-11

**Authors:** Z Mao, X Xu

**Affiliations:** 1 Department of Thoracic Surgery, Renmin Hospital, Wuhan University, Wuhan, Hubei, China; 2 Department of Pharmacology, Tongji Hospital, Huazhong University of Science and Technology, Wuhan, China

## Background

The study is to research if volume-activated chloride channel (VACC) existed in tumor stem cells.

## Methods

CD133+ cells were purified by magnetic cell separation (MACS) system from non-small cell lung cancer (NSCLC) cell line A549. The purity was detected by flow cytometry. VACC was recorded with whole-cell patch clamp.

## Results

The purity of CD133+ cells isolated from A549 by MACS was 92.14%. And VACC was recorded in the 22/40(55%) CD133+ cells of A549 by whole-cell patch clamp.

## Conclusion

VACC exist in CD133+ lung cancer stem cells.

